# 增强型脂质去除净化剂结合超高效液相色谱-串联质谱法测定食品中8种大麻素类化合物

**DOI:** 10.3724/SP.J.1123.2022.08010

**Published:** 2023-05-08

**Authors:** Man SHAO, Xiaoqin YU, Lijuan HUANG, Huan YAO, Shucai LI

**Affiliations:** 四川省食品检验研究院,国家市场监管重点实验室白酒监管技术,四川成都610097; Sichuan Institute of Food Inspection, Key Laboratory of Baijiu Supervising Technology for State Market Regulation, Chengdu 610097, China

**Keywords:** 高效液相色谱-串联质谱, 增强型脂质去除净化剂, 大麻素类化合物, 食品, ultra performance liquid chromatography-tandem mass spectrometry (UPLC-MS/MS), enhanced matrix removal-lipid cleaning adsorbent (EMR-Lipid), cannabinoids, foods

## Abstract

研究在优化前处理条件及色谱分离的基础上,建立了同时测定巧克力、软糖、糕点、饼干、饮料、白酒等6种典型食品基质中8种大麻素类化合物的超高效液相色谱-串联质谱(UPLC-MS/MS)的测定方法。前处理方法中考察了不同的净化方式,采用增强型脂质去除净化剂(EMR-Lipid)解决了巧克力中复杂油脂及软糖中胶质难以去除的问题;同时净化溶液在氮吹前加入10%丙三醇甲醇溶液,解决了目前文献方法中直接氮吹对回收率影响很大的关键问题。样品以乙腈为提取溶剂,EMR-Lipid为净化剂进行净化,无水硫酸钠除水后,乙腈层加入10%丙三醇甲醇溶液100 μL,氮吹至近干;采用Waters ACQUITY UPLC BEH Shield RP18色谱柱进行分离,电喷雾负离子扫描,多反应监测(MRM)模式检测,基质匹配外标法定量。方法学研究表明,四氢大麻酚、次大麻二酚、四氢大麻素、大麻萜酚在2~200 ng/mL范围内线性关系良好;大麻酚、大麻二酚、大麻二酚酸、四氢大麻酚酸A在0.4~40 ng/mL范围内线性关系良好,相关系数(*r*)均大于0.995;检出限和定量限分别为0.8~4 μg/kg和2~10 μg/kg。在不同加标水平下的平均回收率为82.0%~114.9%,相对标准偏差(RSD)均小于15%(*n*=6)。研究结果表明,采用EMR-Lipid净化剂净化食品基质时,净化效果明显,准确度较好。该方法灵敏、快速,准确度高,前处理简单,适用于食品基质中大麻素类化合物的准确定性定量检测。

## Abstract (en)

A novel method was developed for the simultaneous determination of eight cannabinoids in six types of food matrices, including chocolate, fondant, biscuit, beverage, cookie and baijiu, using ultra performance liquid chromatography-tandem mass spectrometry (UPLC-MS/MS). The sample extraction and cleanup steps were optimized, and various purification methods were investigated to remove the oil matrix and glue in chocolate and fudge, respectively. Enhanced matrix removal-lipid adsorbent (EMR-Lipid) provided efficient, selective cleanup of the evaluated matrices. The sample was extracted using acetonitrile, followed by EMR-Lipid cleanup, and then dried using anhydrous sodium sulfate. The acetonitrile layer was concentrated by nitrogen to near-dry after 100 μL 10% glycerol in methanol was added to improve the recovery by reducing loss during concentration under the stream of nitrogen gas. Eight cannabinoids were separated using a Waters ACQUITY UPLC BEH Shield RP18 column (100 mm×3.0 mm, 1.7 μm). The responses of the cannabinoids in the positive and negative ionization modes were investigated and optimized, and the responses were superior in the negative ion mode compared to those in the positive ion mode. MS detection was performed in the multi-reaction monitoring (MRM) mode using an electrospray source in the negative ion mode. The cannabinoids were quantified using an external standard with matrix calibration curves to reduce the influences of the matrix effects on the quantitative results. The developed method was verified, and the conditions of sample pretreatment were also optimized. The calibration curves of tetrahydrocannabinol, cannabidivarin, tetrahydrocannabivarin, and cannabigerol and those of cannabidiol, cannabinol, cannabidiolic acid, and tetrahydrocannabinolic acid exhibited good linearities, with *r*>0.995, in the ranges of 2-200 and 0.4-40 ng/mL, respectively. The respective limits of detection (LODs, *S/N*=3) and quantification (LOQs, *S/N*=10) of tetrahydrocannabinol, cannabidivarin, tetrahydrocannabivarin, and cannabigerol were 4 and 10 μg/kg, and those of cannabidiol, cannabinol, cannabidiolic acid, and tetrahydrocannabinolic acid were 0.8 and 2 μg/kg. The average recoveries of the cannabinoids were 82.0%-114.9% under three spiked levels with corresponding relative standard deviations (RSDs) of <15% (*n*=6). EMR-Lipid provided efficient, selective cleanups of food matrices with good accuracy. The method is sensitive, rapid, accurate, simple to execute, and it is suitable for the determination of cannabinol compounds in typical food matrices.

近几年,北美市场上涌现了大量添加大麻成分的产品,涉及大麻软糖、巧克力、护肤品、饮料、电子烟、酒类及其制品等^[1⇓-3]^,随着我国进出口贸易的逐年增长,一些掺杂了大麻的食品也进入国内。近几年,海口、广州及苏州海关在例行查验时也发现了含有大麻成分的巧克力、饼干、软糖等部分食品^[4]^。目前我国仍将大麻列为毒品进行严格管制,但暂未制定含有大麻成分的食品的相关法律法规、限量标准和检测标准。为杜绝含有大麻成分的食品出现在国内市场,同时为了监管国内外工业大麻食品的安全性,相关食品基质中大麻素类化合物的检测尤为重要。

大麻素(cannabinoids)及其衍生物是大麻植物中特有的一类含有烷基及单萜分子结构的次生代谢产物^[5]^,具有代表性的化合物主要包括四氢大麻酚(tetrahydrocannabinol, THC)、大麻二酚(cannabidiol, CBD)、大麻酚(cannabinol, CBN)、大麻二酚酸(cannabidiolic acid,CBDA)、次大麻二酚(cannabidivarin, CBDV)、四氢大麻酚酸A(tetrahydrocannabinolic acid, THCA-A)、四氢大麻素(tetrahydrocannabivarin, THCB)、大麻萜酚(cannabigerol, CBG)等,结构信息见图1。

**图1:**
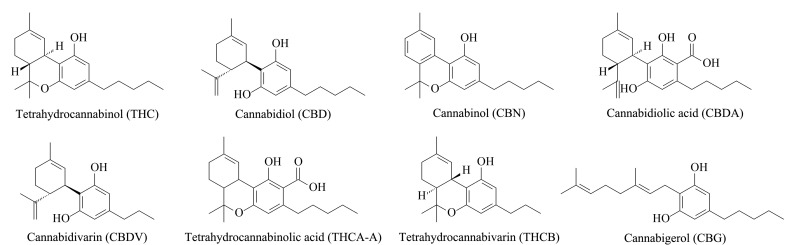
8种大麻素类化合物的结构信息

THC是大麻中的主要精神活性物质,具有镇痛、抗炎、抗惊厥、刺激食欲等作用^[6,7]^,但THC会使人产生幻觉。CBD可以抗抑郁、抗焦虑,并对心血管系统和呼吸系统具有保护作用。近些年酸性大麻素也被报道具有潜在的治疗价值^[8⇓⇓-11]^,但目前我国将大麻素类物质列为毒麻药品并禁止在食品工业中使用。2018年,美国分析化学协会(AOAC)在征集大麻素类物质的检测方法^[12]^,该方法要求目标检测物包含THC、CBD、CBN、CBDA、CBDV、THCA-A、THCB、CBG等物质,说明食品基质中此8种大麻素的同时检测十分必要。

根据文献报道,目前检测大麻素类物质的方法主要包括气相色谱法(GC)^[13]^、高效液相色谱法(HPLC)^[14,15]^、气相色谱-质谱联用法(GC-MS)^[16]^、高效液相色谱-串联质谱法(HPLC-MS/MS)^[17,18]^等。相关研究的前处理多数是采用溶剂直接萃取,未对提取溶液进行很好的净化处理。少部分文献虽然对提取液进一步用固相萃取柱或者其他的净化方式进行净化,但食品基质中化学成分复杂,净化液中仍含有一定量的胶质、高级脂肪酸酯等成分,会影响待测组分测定结果的准确性。本研究发现采用增强型脂质去除净化剂(EMR-Lipid)可有效去除复杂食品基质中的胶质、高级脂肪酸酯等成分,准确度较为理想。

目前,国内大麻素类物质的检测标准主要有公安行业标准^[19]^及司法鉴定规范^[20]^,主要是针对THC、CBD、CBN这3种化合物;大麻植株^[21]^、火麻油^[22,23]^、化妆品^[24]^、药品^[25]^、人体毛发^[26]^、血液^[27,28]^以及尿液^[29]^等基质中THC、CBD、CBN的研究也较多。但检测相关食品基质中大麻素及其衍生物的文献较少,且均无现行有效的标准方法。结合我国国情,同时为了满足进出口检验的需要,建立一种兼顾高效性、高分辨率、高准确度的典型食品基质中大麻素类化合物的分析方法非常必要。本文对照主要国家对食品基质中大麻的限量要求及我国进出口现状^[4]^,选取巧克力、软糖、糕点、饼干、饮料及白酒这6种具有代表性的食品基质,建立了超高效液相色谱-串联质谱法测定THC、CBD、CBN、CBDA、THCA-A、CBDV、THCB、CBG的方法,为相关产品的安全监管及风险防范奠定了技术基础。

## 1 实验部分

### 1.1 仪器、试剂与材料

6500超高效液相色谱-串联质谱仪(美国SCIEX公司,配岛津LC-30AD); MS3 digital涡旋振荡器(美国IKA公司); FB15065超声波清洗器、X3R高速冷冻离心机(美国Fisher公司); ME204电子天平(美国梅特勒公司); N1-50氮吹仪(上海屹尧有限公司); Milli-Q纯水仪(德国默克公司)。

甲醇、乙腈(色谱纯,中国KNOWLES公司);乙酸铵(色谱纯,美国Fisher公司);丙三醇(分析纯,成都市科龙化工试剂厂);无水硫酸钠(分析纯,成都金山化学试剂有限公司);氯化钠(分析纯,成都市科隆化学品有限公司); EMR-Lipid净化剂(部件号: 5982-1010,美国Agilent公司)。

THC、CBD、CBN、CBDA、CBDV、THCA-A、THCB、CBG标准溶液(100 mg/L)均购自天津阿尔塔科技有限公司。

巧克力、软糖、饼干、饮料、糕点、白酒为市售或网购。

### 1.2 实验条件

#### 1.2.1 标准溶液的配制

混合标准中间液:分别精密吸取CBD、CBN、CBDA、THCA-A标准溶液各0.2 mL, THC、CBDV、THCB、CBG标准溶液各1 mL,置于同一10 mL容量瓶中,用乙腈定容至刻度,混匀,制成混合标准储备液(CBD、CBN、CBDA、THCA-A质量浓度为2 mg/L, THC、CBDV、THCB、CBG质量浓度为10 mg/L)。

标准系列工作溶液:用空白基质溶液将混合标准中间液稀释成质量浓度分别为0.4、1、2、4、10、20、40 μg/L的CBD、CBN、CBDA、THCA-A系列标准溶液;质量浓度分别为2、5、10、20、50、100、200 μg/L的THC、CBDV、THCB、CBG系列标准溶液。

#### 1.2.2 样品前处理

称取1 g(精确至0.01 g)已混匀样品于50 mL具塞离心管中,加入10 mL乙腈,涡旋混合30 s。超声提取15 min, 4 ℃下以10000 r/min离心5 min,将乙腈层移入另一洁净试管中待净化。EMR-Lipid净化剂使用前先用3 mL水活化,加入上述上清液,涡旋混合2 min,在4 ℃下以8000 r/min离心5 min,上清液移入已装有1 g氯化钠和1 g无水硫酸钠的离心管中,涡旋混合2 min, 4 ℃下以10000 r/min离心5 min,精密吸取5 mL乙腈层于氮吹管中,再精密加入100 μL 10%丙三醇甲醇溶液,于40 ℃氮气浓缩至近干,精密加入甲醇1 mL,涡旋30 s,超声1 min,过0.22 μm滤膜,待测定。

#### 1.2.3 HPLC-MS/MS检测条件

色谱柱:Waters ACQUITY UPLC BEH Shield RP18色谱柱(100 mm×3.0 mm, 1.7 μm);流动相:A为10 mmoL/L乙酸铵溶液,B为甲醇。梯度洗脱程序:0~9.0 min, 65%B~100%B; 9.0~9.1 min, 100%B~65%B; 9.1~12.0 min, 65%B。柱温:30 ℃;流速:0.3 mL/min;进样量:5 μL。

离子化模式:电喷雾电离,负离子模式(ESI^-^);质谱扫描方式:多反应监测(MRM);气帘气压力(CUR): 55 kPa;碰撞气压力(CAD): 35 kPa;喷雾电压(IS): -4500 V;离子源温度(TEM): 500 ℃;雾化气压力(GS1): 207 kPa;辅助加热气压力(GS2): 207 kPa;射入电压(EP): 10 V;碰撞室射出电压(CXP): 11 V。其他质谱参数,包括定量与定性离子对、去簇电压(DP)及碰撞能量(CE)见表1。

**表1:** 8种大麻素类化合物的主要质谱参数

Compound	Precursor ion (*m/z*)	Product ion (*m/z*)	DP/V	CE/eV	Compound	Precursor ion (*m/z*)	Product ion (*m/z*)	DP/V	CE/eV
THC	313.0	245.0^*^	-142	-34	CBDV	285.0	217.2^*^	-100	-38
		191.0		-37			106.9		-38
CBD	313.1	245.1^*^	-100	-32	THCA-A	357.0	313.2^*^	-100	-38
		179.0		-27			245.1		-40
CBN	309.1	279.1^*^	-150	-40	THCB	284.9	217.1^*^	-100	-36
		222.0		-60			163.0		-34
CBDA	357.1	339.3^*^	-5	-36	CBG	315.1	135.9^*^	-121	-38
		245.1		-38			190.9		-34

DP: declustering potential; CE: collision energy; * quantitative ion.

## 2 结果与讨论

### 2.1 实验条件的考察

#### 2.1.1 仪器条件的优化

根据化合物的结构特性及文献报道^[13⇓⇓⇓⇓-18]^,研究首先考察了Waters ACQUITY UPLC BEH C18(100 mm×2.1 mm, 1.7 μm)、Thermo ACE Excel C18(75 mm×2.1 mm, 1.8 μm)、Agilent Eclipse Plus C18(100 mm×2.1 mm, 1.8 μm)、Agilent XDB C18(150 mm×3.0 mm, 1.8 μm)、Phenomenex Kinetex F5(50 mm×3.0 mm, 2.6 μm)、Waters AQ C18(100 mm×2.1 mm, 1.7 μm)等色谱柱。研究结果表明,在以上6种色谱柱中,THCB、CBDV、CBD及THC、THCA-A、CBN均较难分离。经过调研,Waters ACQUITY UPLC BEH Shield RP18色谱柱(100 mm×3.0 mm, 1.7 μm)在键合相中加入了氨基甲酸酯基团,可以改善碱性、酚类、酚酸类、多羟基类化合物的峰形及响应,因此相比于普通直链C18,更加适合酚类化合物的分析。研究过程中发现,采用Waters ACQUITY UPLC BEH Shield RP18色谱柱时,8种大麻素类化合物峰形较好,分离效果最佳(见图2),因此最终选择Waters ACQUITY UPLC BEH Shield RP18色谱柱进行后续的实验研究及方法验证。

**图2:**
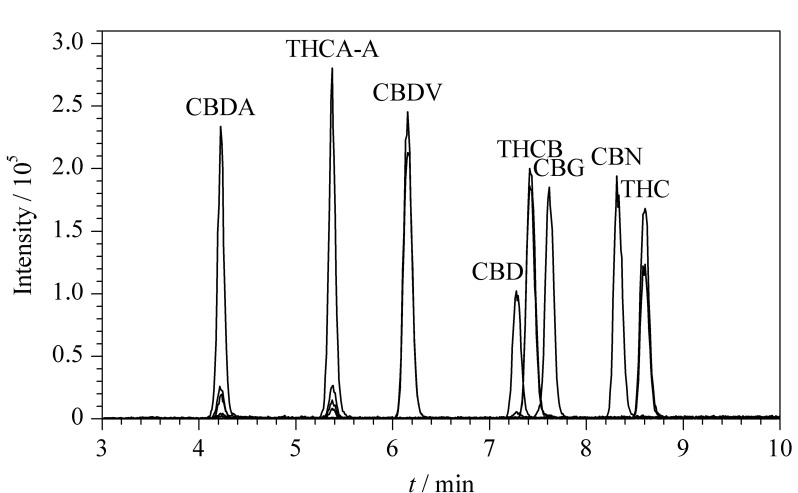
8种大麻素类化合物的总离子流图

本研究还对流动相的组成进行了考察,比较了用甲醇、乙腈作为有机相,0.1%甲酸、10 mmol/L乙酸铵溶液、10 mmol/L乙酸铵溶液(含0.1%甲酸)作为水相的情况。最终确定流动相的组成为甲醇和10 mmol/L乙酸铵溶液。

#### 2.1.2 提取溶剂的选择

大麻素类化合物难溶于水,溶于多种有机溶剂。EMR-Lipid净化剂推荐使用乙腈或者丙酮作为提取溶剂,不推荐使用与水不互溶的溶剂以及醇类等。实验采用空白基质加标的形式,以基质较为复杂的巧克力作为典型基质,对比了乙腈和丙酮作为提取溶剂时各化合物的回收率,分别为82.5%~105.6%和86.8%~103.1%。结果显示,两者的提取效率无显著差别,但丙酮属于易制毒试剂,受公安部门管制,因此实验过程中选择乙腈作为提取溶剂。

#### 2.1.3 净化方式的选择

通过查阅文献[13⇓⇓⇓⇓-18],实验采用空白基质加标的形式,以巧克力、软糖、糕点、饼干、饮料及白酒为基质,首先根据目标化合物的结构和研究基质的特点考察了不同的QuEChERS试剂,主要包括QuEChERS 1(*N*-丙基乙二胺)、QuEChERS 2(硅胶键合*N*-丙基乙二胺+C18+柠檬酸钠+柠檬酸二钠)、QuEChERS 3(中性氧化铝+硅酸镁+石墨化炭黑)。结果表明,上述试剂对目标化合物有不同程度的吸附,回收率仅为24.3%~59.6%,且巧克力中的油脂、软糖中的胶质无法除去。其次,根据目标化合物的结构和保留机理,考察了不同的固相萃取柱,主要包括二乙烯苯-*N*-乙烯基吡咯烷酮固相萃取柱(HLB)、石墨化炭黑/氨基柱(Carb/NH_2_)、中性氧化铝小柱(Alumina-N)、弗罗里硅土固相萃取柱(Florisil)、聚苯乙烯/二乙烯苯固相萃取柱(PEP)、PRiME HLB固相萃取柱等。结果表明,HLB及Carb/NH_2_对待测组分基本无保留,Alumina-N、Florisil、PRiME HLB这3种固相萃取柱的回收率分别为20.1%~60.8%、35.8%~58.7%、59.4%~71.3%,巧克力中的油脂、软糖中的胶质仍无法除去;用PEP净化的平均回收率为69.8%~90.6%,较之前的净化方式有明显提高,但仍不能有效除去巧克力中的油脂,复溶液放置1~2 h内产生白色沉淀,净化效果仍不理想。最后根据目标化物的结构和基质的复杂性,考察了EMR-Lipid这一增强型脂质去除净化剂,EMR-Lipid净化剂使用化学键合相,为一种特殊聚合物基质,通过体积排阻和疏水相互作用的组合机制,专门吸附脂质中C5及以上碳链的化合物,可显著改善脂质去除的选择性,减少净化过程中疏水性化合物的损失。结果表明,应用EMR-Lipid净化,平均回收率为89.5%~101.5%,能显著去除复杂食品基质中的胶质、高级脂肪酸酯,且复溶液放置12 h后无沉淀产生,净化效果和回收率均最好。实验过程中确定采用EMR-Lipid净化食品基质。不同净化方式的回收率详细信息见图3。

**图3:**
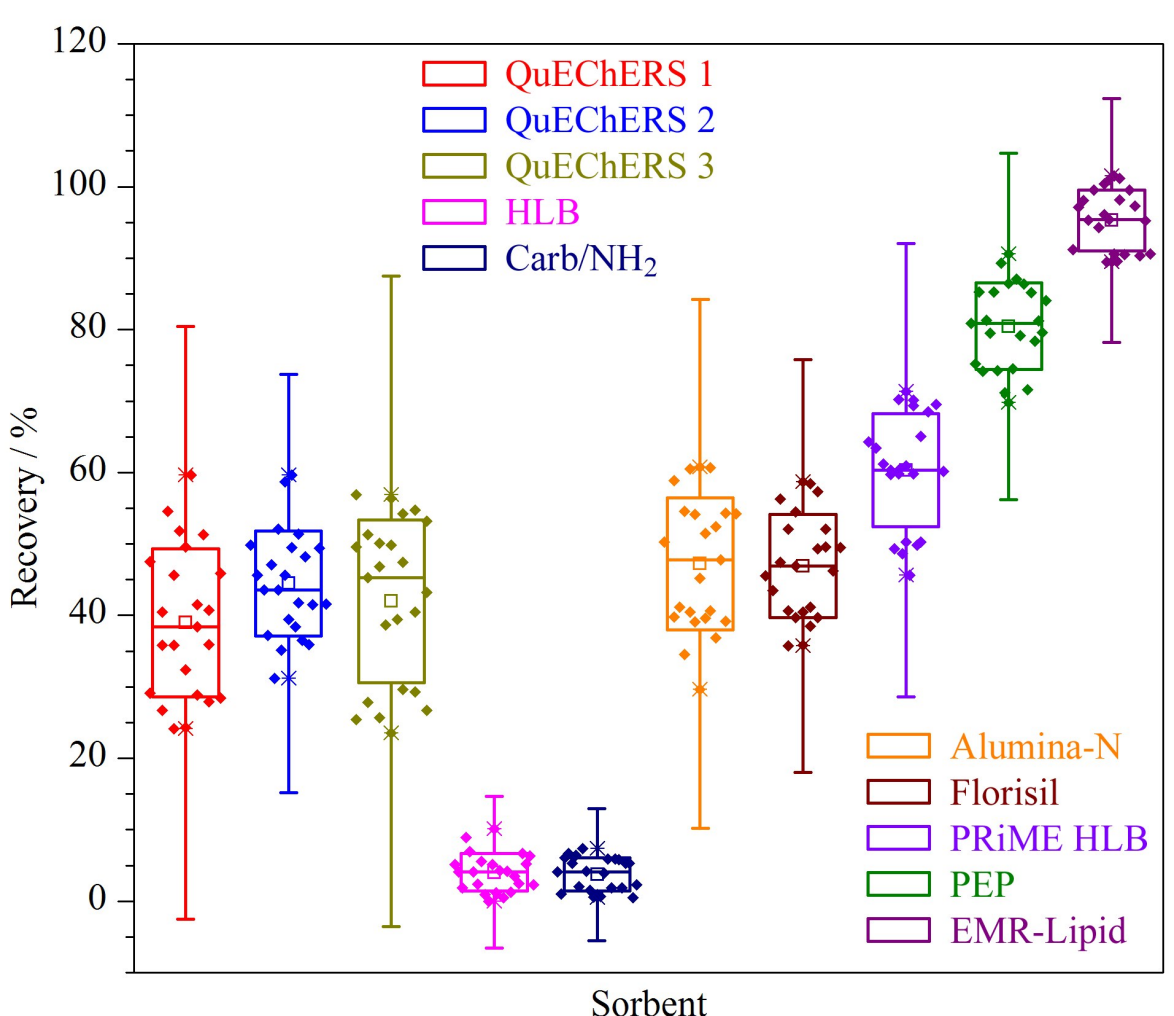
不同净化方式对8种大麻素类化合物回收率的影响

#### 2.1.4 氮吹的影响因素

本研究选择氮吹进行乙腈提取溶液的浓缩。首先考察了8种大麻素类化合物的标准溶液在40 ℃直接氮吹至近干后的回收率,8种大麻素化合物的平均回收率范围为60.3%~95.4%,其中THC、CBD、CBN、THCB、CBG的回收率较低,可见直接氮吹会造成待测组分不同程度的损失。为提高氮吹过程中的保留,经查阅文献[30,31],考虑在氮吹前加入二甲基亚砜(DMSO)或者10%丙三醇甲醇溶液以提高回收率。DMSO和丙三醇沸点较高,与乙腈互溶,并对各种化合物具有良好的溶解性;通过在氮吹前加入微量的DMSO或者丙三醇,可使待测物保留在上述两种溶剂中,减少氮吹过程中的损失,有效提高待测物的回收。研究过程中,使用空白基质加标的方式,以基质较为复杂的巧克力为典型基质,比较了①氮吹前加入100 μL 10%丙三醇甲醇溶液、②氮吹前加入100 μL DMSO和③直接氮吹至近干时大麻素类化合物的回收率,结果见图4。结果表明,氮吹前加入100 μL DMSO和100 μL 10%丙三醇甲醇溶液均能明显提高巧克力中大麻素类化合物的回收率,其中氮吹前加入10%丙三醇甲醇溶液使8种大麻素类化合物的回收率提高更为显著,原因可能是在氮吹至近干的过程中,DMSO聚集在氮吹管最底部,而10%丙三醇甲醇溶液则在氮吹管下部形成湿润的液膜层,可使化合物在加热氮吹过程中保留在液膜层中。综合考虑,确定在氮吹前加入100 μL 10%丙三醇甲醇溶液以改善回收率。

**图4:**
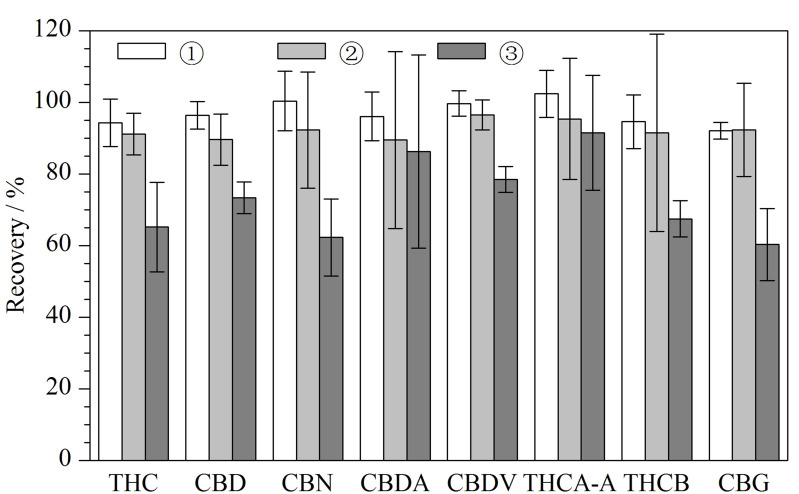
采用不同氮吹方式时8种大麻素类化合物的回收率(*n*=3)

### 2.2 基质效应

由于质谱仪响应较易受到样品基质的干扰,实验过程中采用相对响应值法对巧克力、软糖、饼干、饮料、糕点、白酒中的基质效应(ME)进行了考察,即通过计算基质匹配标准曲线与纯溶剂标准曲线的斜率比来评价方法的基质效应^[30]^。一般来说,当基质效应值在80%~120%之间时,表明基质效应在可接受范围内,在实际测定时可以忽略基质效应;反之则应考虑基质效应对实际测定时的影响。6种基质对8种化合物的ME计算结果见表2。结果显示,6种基质中8种大麻素类化合物均存在不同程度的基质效应,因此,实验过程中需采用基质匹配标准曲线来校正,以降低基质效应对定量结果的影响。

**表2:** 8种大麻素类化合物在6种基质中的基质效应

Compound	Chocolate	Fondant	Biscuit	Beverage	Cookie	Baijiu
THC	16.6	31.8	7.5	37.9	25.0	69.6
CBD	34.5	75.8	22.5	60.6	54.4	75.3
CBN	64.7	58.9	18.6	60.8	43.3	78.1
CBDA	111.9	39.4	74.3	34.7	48.9	75.8
CBDV	77.3	68.5	89.1	59.0	75.9	80.4
THCA-A	85.9	31.5	71.0	28.5	62.8	77.6
THCB	136.9	50.4	16.4	50.8	39.7	72.8
CBG	19.5	57.1	17.8	54.7	43.5	69.4

### 2.3 方法学考察

#### 2.3.1 线性范围和相关系数

分别采用6种空白基质配制标准系列溶液,在已优化的色谱与质谱条件下,以峰面积(*y*)对相应的质量浓度(*x*, μg/L)作图,绘制标准曲线,并计算各待测物线性回归方程及其相关系数。结果表明,CBD、CBN、CBDA、THCA-A在0.4~40 μg/L范围内,THC、CBDV、THCB、CBG在2~200 μg/L范围内,8种大麻素类化合物的线性关系良好,相关系数(*r*)均在0.995以上。

#### 2.3.2 检出限及定量限

采用空白基质加标并逐级稀释的方式进行检出限(LOD)和定量限(LOQ)的确定,按1.2.2节的方法处理后,采用1.2.3节的条件进行检测。根据各化合物的特征色谱峰的信噪比(*S/N*)=3和*S/N*=10时对应的加标水平确定LOD和LOQ,最终确定THC、CBDV、THCB、CBG的检出限均为4 μg/kg,定量限均为10 μg/kg, CBD、CBN、CBDA、THCA-A的检出限均为0.8 μg/kg,定量限均为2 μg/kg。

#### 2.3.3 回收率和精密度

按照前述方法,对不含待测物的巧克力、软糖、饼干、饮料、糕点、白酒等6种空白样品进行加标回收试验,CBD、CBN、CBDA、THCA-A加标水平分别为2、5、20 μg/kg, THC、CBDV、THCB、CBG加标水平分别为10、25、100 μg/kg,各水平制样6份,考察方法的准确度和精密度,结果见表3。结果表明,8种大麻素类化合物的平均回收率为82.0%~114.9%,相对标准偏差(RSD, *n*=6)为1.2%~12.3%,方法准确度和精密度良好,能满足分析要求。

**表3:** 8种大麻素类化合物的加标回收率和相对标准偏差(*n*=6)

No.	Compound	Spiked/ (μg/kg)	Chocolate			Fondant			Biscuit			Beverage			Cookie			Baijiu		
			Recovery/ %	RSD/ %		Recovery/ %	RSD/ %		Recovery/ %	RSD/ %		Recovery/ %	RSD/ %		Recovery/ %	RSD/ %		Recovery/ %		RSD/ %
1	THC	10	104.6	3.3		89.5	9.8		94.8	4.8		84.2	10.8		100.4	2.9		83.2		9.8
		25	99.4	2.1		82.3	5.1		89.8	3.6		89.3	5.8		99.2	1.7		90.4		5.6
		100	104.6	4.3		87.2	3.9		100.1	3.2		95.2	3.6		94.9	5.9		92.4		2.5
2	CBD	2	106.0	9.1		88.1	10.3		98.8	5.4		86.4	1.6		100.8	3.2		88.6		5.8
		5	104.1	2.6		85.4	5.2		89.8	5.9		86.0	4.5		94.7	6.1		88.9		5.3
		20	114.9	6.1		86.5	6.4		88.7	5.3		94.2	5.0		96.6	8.6		95.2		2.1
3	CBN	2	100.8	9.2		91.1	8.7		93.1	5.0		85.3	8.1		88.4	9.9		90.3		6.1
		5	96.8	8.1		88.2	8.1		96.4	4.4		89.4	5.5		91.5	3.1		95.1		2.6
		20	90.0	6.7		92.9	6.0		103.3	3.8		89.0	4.8		88.6	5.0		92.6		4.3
4	CBDA	2	106.1	3.4		89.7	5.9		90.4	10.0		98.3	4.2		92.9	6.5		95.8		2.8
		5	102.9	8.9		84.0	9.5		95.6	8.8		107.8	1.7		96.5	4.3		98.7		1.8
		20	89.8	6.7		86.7	4.7		89.0	7.0		106.7	8.0		88.3	3.8		100.2		3.6
5	CBDV	10	94.9	5.1		88.1	7.6		86.3	3.4		89.1	3.4		92.0	12.3		92.1		1.9
		25	82.0	5.9		87.3	6.2		83.1	1.5		88.1	9.5		96.0	2.6		94.6		5.8
		100	95.0	7.0		85.4	3.3		89.5	3.8		91.1	3.9		96.1	2.8		92.5		1.7
6	THCA-A	2	102.5	5.5		87.5	9.4		92.4	8.8		86.1	6.7		94.6	8.8		89.5		6.9
		5	103.3	11.1		85.3	6.1		92.3	7.5		89.3	7.5		92.3	7.5		93.2		4.1
		20	103.1	9.0		86.7	6.3		87.2	4.3		92.7	5.5		87.2	4.3		96.4		5.3
7	THCB	10	100.4	3.4		88.8	8.1		97.3	1.2		100.2	3.0		97.3	2.2		99.2		8.1
		25	100.8	2.1		85.0	3.1		95.2	4.4		91.4	9.1		95.2	4.4		95.7		9.7
		100	101.4	2.6		83.9	5.7		90.3	8.7		95.4	4.0		90.3	8.7		99.6		1.6
8	CBG	10	107.2	5.8		89.4	8.7		101.0	6.8		91.4	2.4		101.0	6.8		91.7		2.5
		25	95.9	4.2		88.9	7.5		99.4	3.6		89.5	5.2		99.4	3.6		92.3		3.5
		100	102.8	2.3		90.4	7.0		96.4	2.9		97.4	4.4		96.4	2.9		99.7		1.9

### 2.4 实际样品测定

采用本实验建立的方法,对从市场购买和网购的60个样本进行测定,其中巧克力12批、软糖10批、饼干10批、饮料10批、糕点8批、白酒10批,均未检出8种大麻素类化合物。

## 3 结论

本文建立了UPLC-MS/MS测定巧克力等6种基质中8种大麻素类化合物的方法。该方法的回收率及精密度良好,重复性较好。方法前处理较为简单快速,准确度和灵敏度较高,可作为快速的定性和定量的分析方法,为大麻素类化合物后续的研究奠定了基础,也为我国开展食品基质中大麻素类化合物的安全性评价提供了技术支撑。
